# Supplementary material for: Combined comparative genomics and clinical modeling reveals plasmid-encoded genes are independently associated with Klebsiella infection
Source: Nat Commun. 2022 Aug 1;13:4459. doi: 10.1038/s41467-022-31990-1 (PMC9343666; doi:10.1038/s41467-022-31990-1)
Supplement: Supplementary file 1 — Supplementary Information [file 41467_2022_31990_MOESM1_ESM.pdf]

Supplementary information

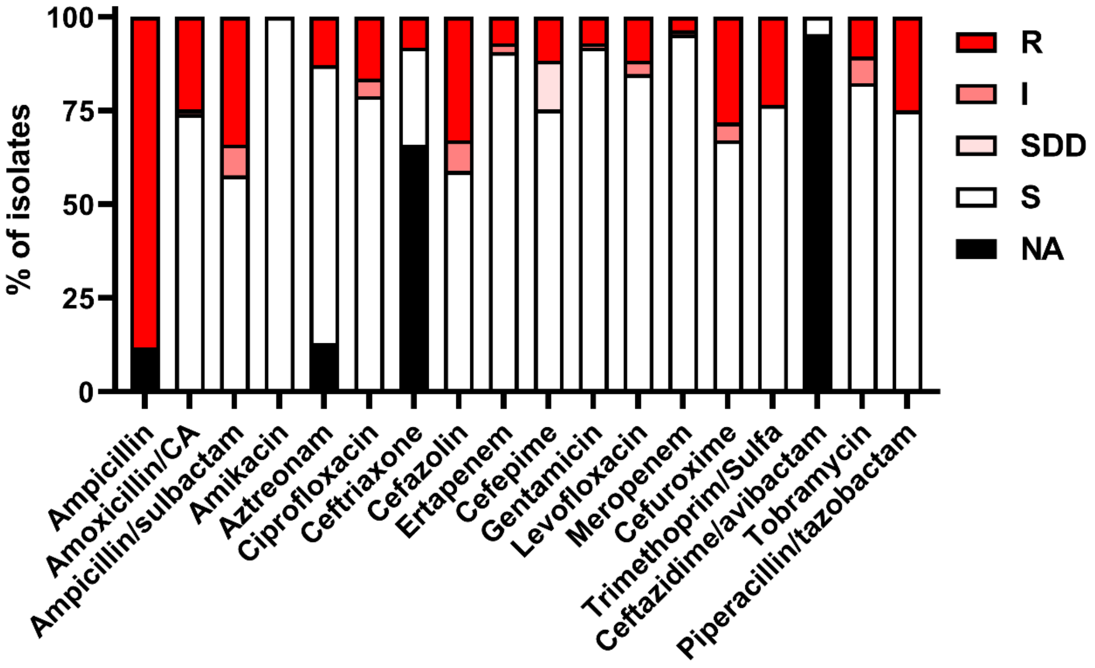

Supplementary Figure S1. Antibiotic susceptibility results from *Klebsiella* clinical isolates.

Categorical results of antibiotic susceptibility testing by broth microdilution (n = 85). R = Resistant, I = Intermediate, SDD = Susceptible-Dose-Dependent, S = Sensitive.

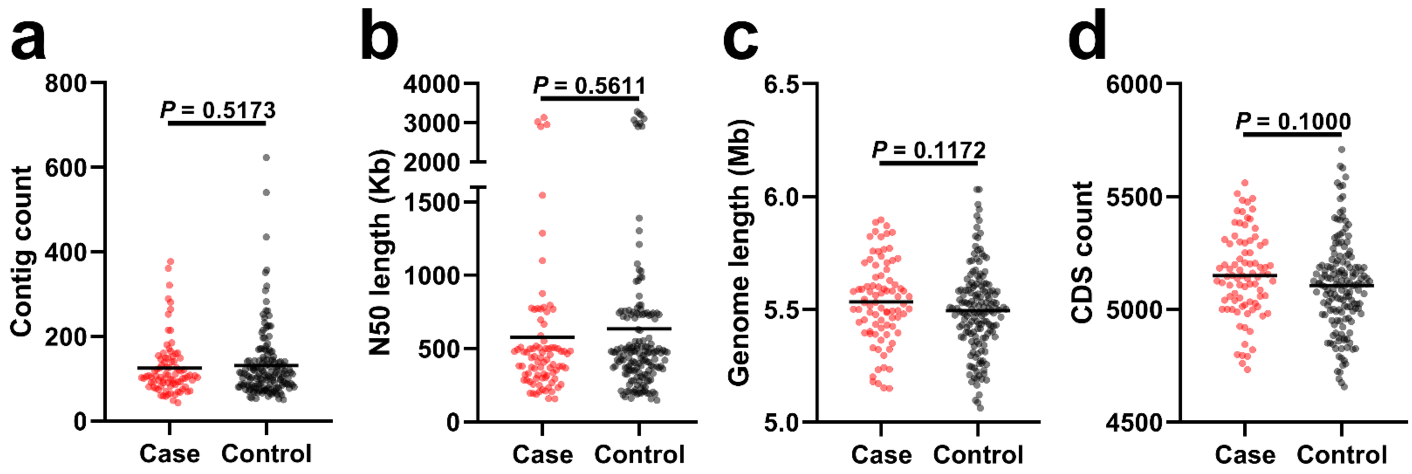

Supplementary Figure S2. *Klebsiella* genome assembly details compared between case and control rectal isolates.

a Comparison of number of contigs for *Klebsiella* genome assemblies between cases (n = 85) and controls (n = 160, mean displayed, two-sided Student's t-test). **b** Comparison of N50 length for *Klebsiella* genome assemblies between cases (n = 85) and controls (n = 160, mean displayed, two-sided Student's t-test). **c** Comparison *Klebsiella* genome length between cases (n = 85) and controls (n = 160, mean displayed, two-sided Student's t-test). **d** Comparison of number of coding sequences (CDS) for *Klebsiella* genome assemblies between cases (n = 85) and controls (n = 160, mean displayed, two-sided Student's t-test). For **a**-**d**, each data point represents a single *Klebsiella* genome assembly.

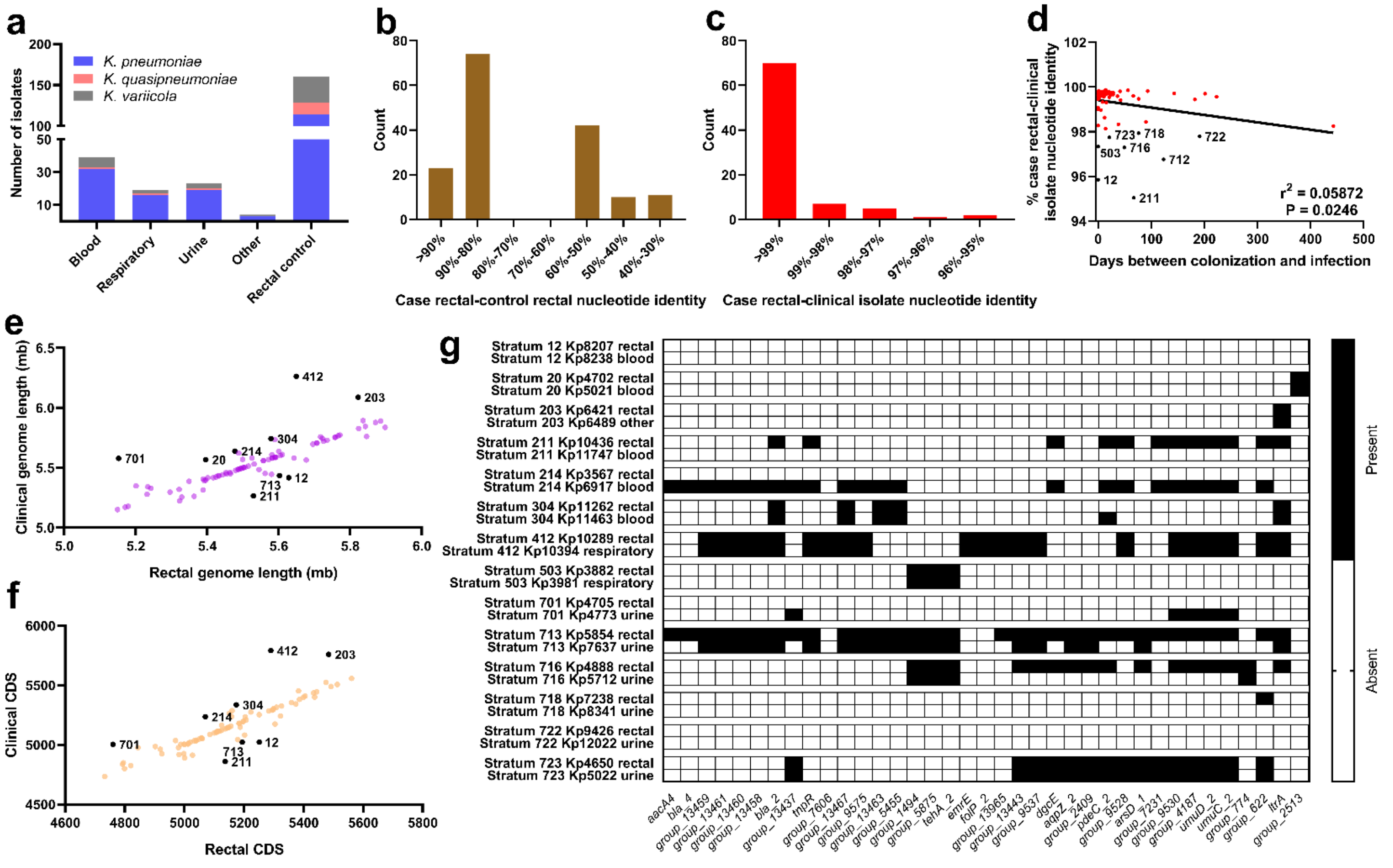

Supplementary Figure S3. *Klebsiella* genome alignments.

a *Klebsiella* species distribution across infection and colonization sites. **b** Histogram of case rectal-control rectal nucleotide alignment. **c** Histogram of case clinical-case rectal nucleotide alignment. **d** Correlation between the number of days between identification of a rectal *Klebsiella* isolate concordant with the clinical isolate from the same patient and case clinical-case rectal nucleotide alignment (Spearman correlation, two-sided). **e** Plot of case clinical genome length and case rectal genome length. **f** Plot of number of annotated coding sequences (CDS) and genome length for each rectal case genome. For **d**-**f**, strata that diverge from overall data trends are highlighted by black data points and indicated by their respective stratum number. **g** Heatmap of presence/absence of infection-associated genes for strata that diverge from overall data trends in **d**-**f**.

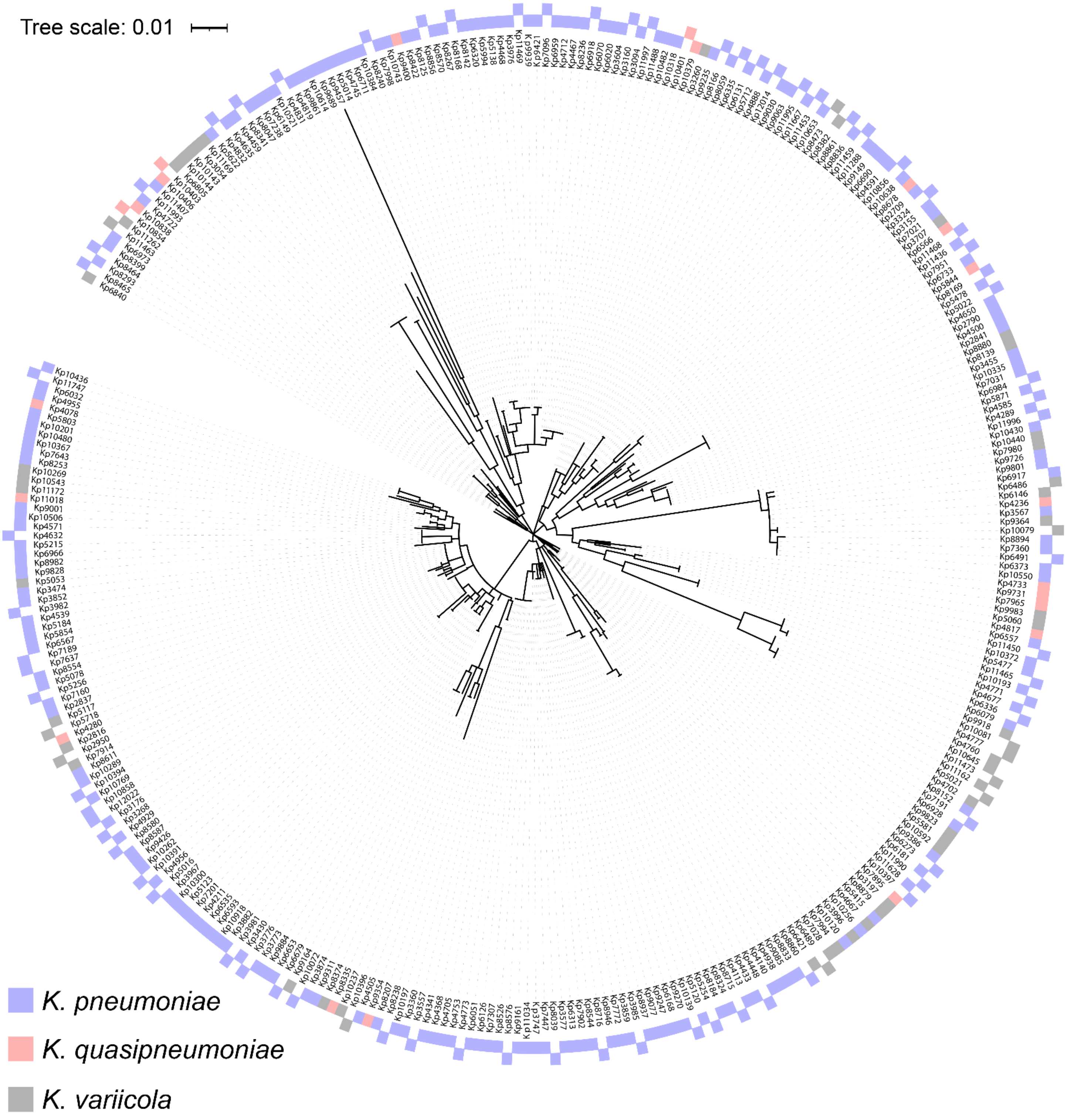

Supplementary Figure S4. *wzi* phylogeny of sequences *Klebsiella* isolates.

Phylogenetic tree of sequenced *Klebsiella* rectal isolates based on *wzi* sequence. Filled rectangles on the inner track indicate a rectal isolate and filled rectangles on the outer track indicate a clinical isolate.

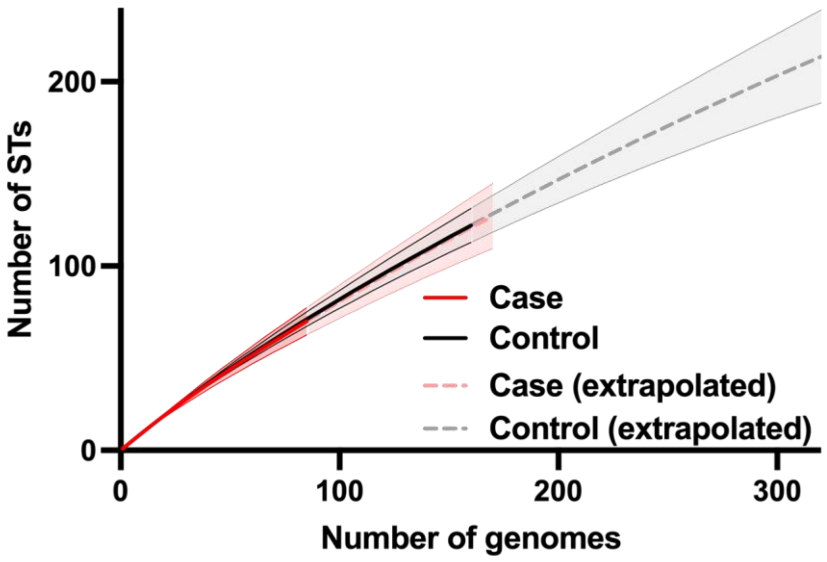

Supplementary Figure S5. Rarefaction of *Klebsiella* sequence types.

There was no difference in the richness of sequence types (STs) between cases and controls, when compared at 85 (the number of cases) or when cases were extrapolated to 160 (the number of controls). The estimated asymptotes of these curves do not differ (Case = 529.653 ± 259.103, Control = 591.953 ± 170.906 [mean ± SE]).

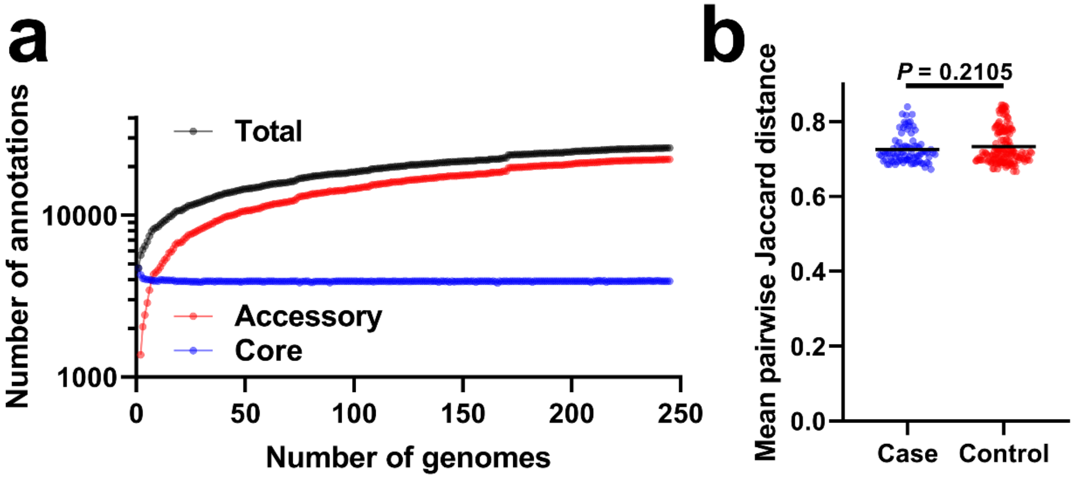

Supplementary Figure S6. Characterization of pangenome annotation

a Cumulative count of *Klebsiella* genome annotations stratified by core and accessory genome. **b** Mean pairwise Jaccard distances were calculated for all rectal isolates and compared between cases (n = 85) and controls (n = 160, mean displayed, two-sided Student's t-test). Each data point represents an individual isolate.

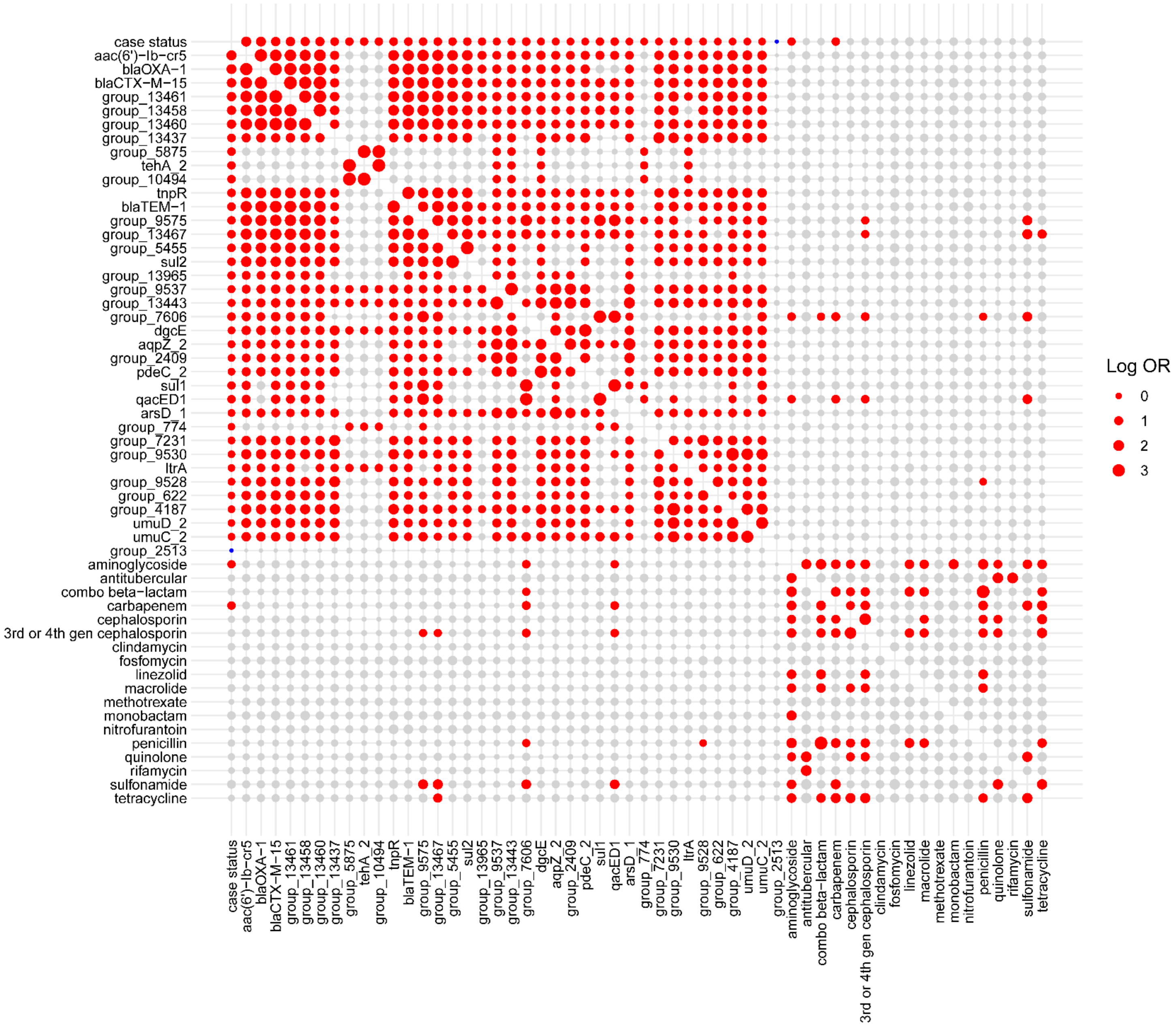

Supplementary Figure S7. Associations within and between infection-associated genes and prior exposure to individual antibiotics.

Odds ratios and Fisher *P* values (two-sided) were calculated between all pairs of infection-associated genes and prior antibiotics. Log Odds ratios displayed in red and blue have a Fisher *P* value ≤ 0.01. *group_2513* is shown in blue to highlight its negative association with case status.

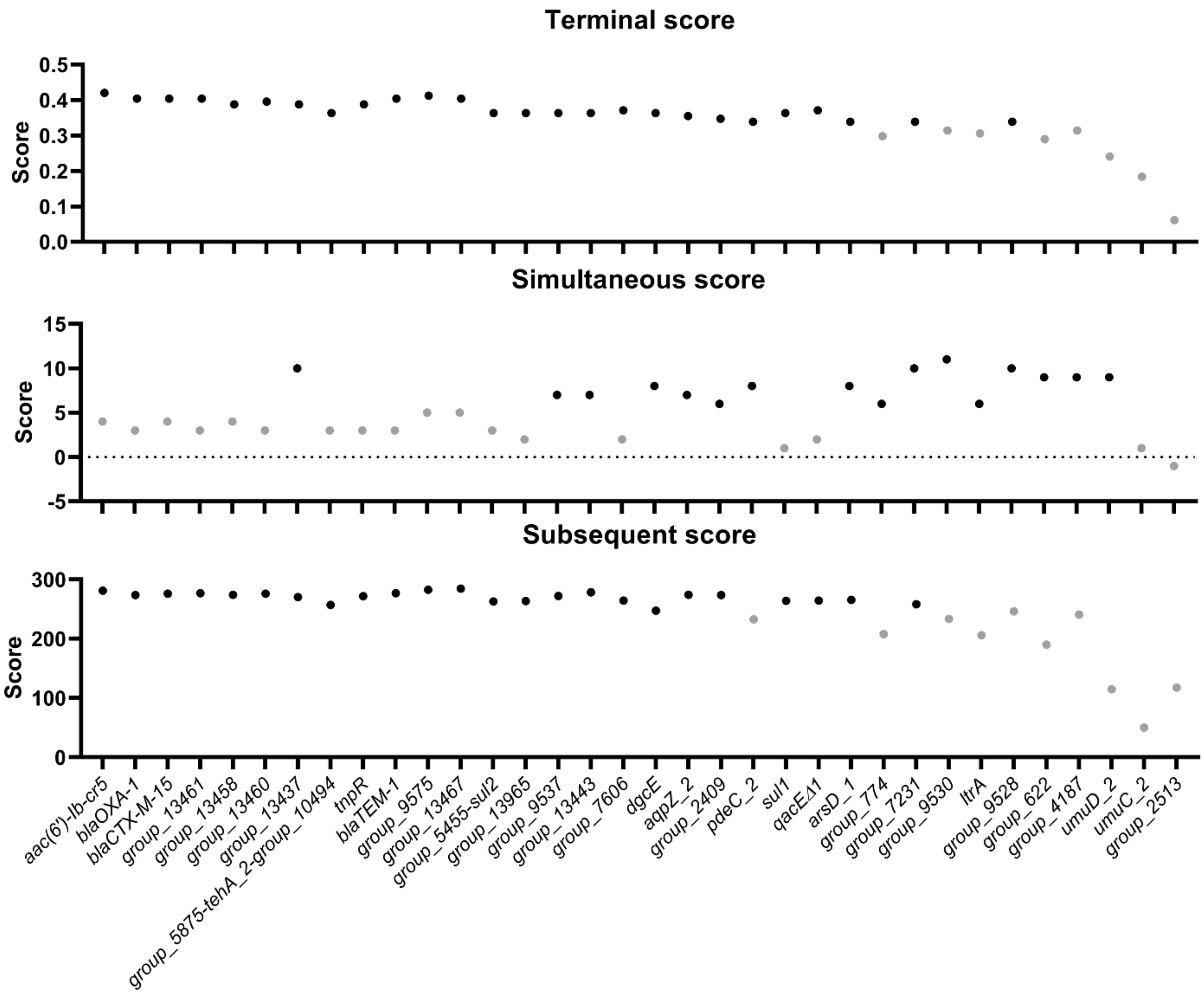

Supplementary Figure S8. Evaluation of association between gene presence and infection using treeWAS

Manhattan plots for the “Terminal score,” “Simultaneous score,” and “Subsequent score” outputs from treeWAS applied to the 34 infection-associated loci. The Terminal score counts the four possible combinations of genotype and phenotype without regard to phylogeny. The Simultaneous and Subsequent scores are based on the number of branches with simultaneous changes in genotype and phenotype, and the proportion of the tree where the phenotype and genotype co-exist, respectively, and account for population structure. Black symbols indicate a *P* value ≤ 0.01, and grey symbols indicate a *P* value > 0.01. Statistical testing is two-sided and not corrected for multiple comparisons. Raw values can be found in Supplementary Table 1.

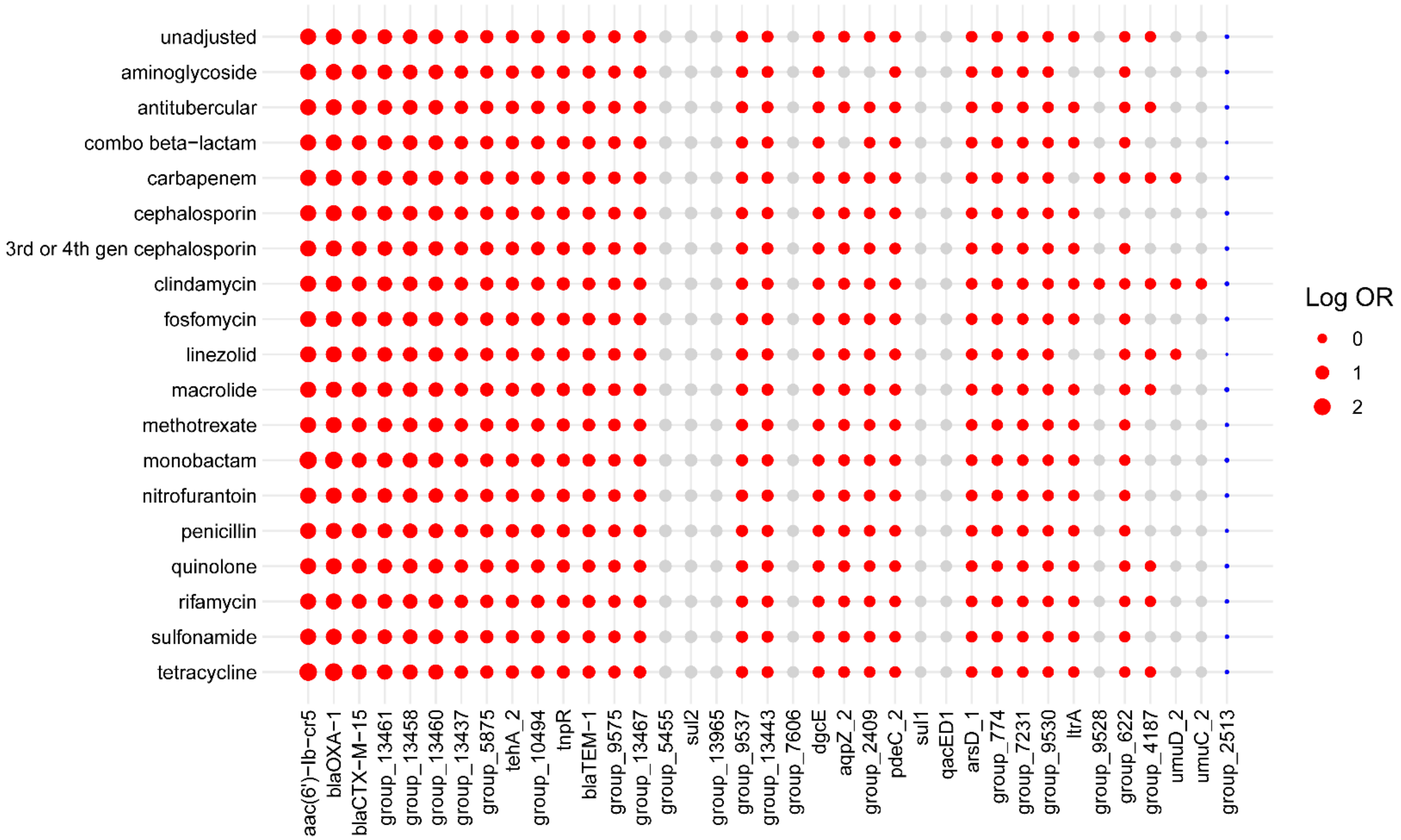

Supplementary Figure S9. Adjustment of association between gene presence and infection for prior exposure to individual antibiotics

The association of each gene (columns) was adjusted for prior exposure to each antibiotic (rows). Log Odds ratios displayed in red and blue have an adjusted Fisher *P* value ≤ 0.01. *group_2513* is shown in blue to highlight its negative association with case status. Statistical testing is two-sided and not corrected for multiple comparisons.

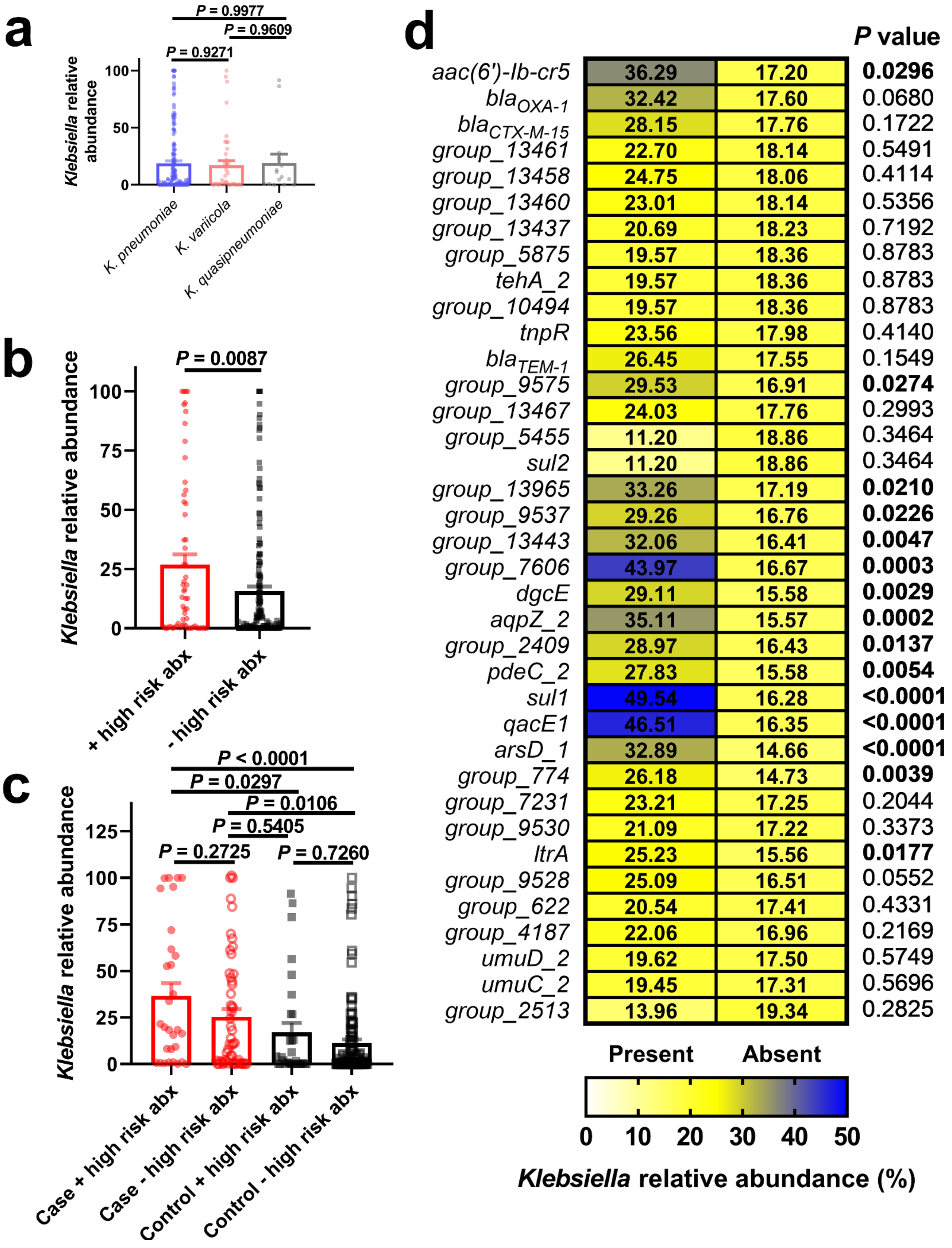
a
P = 0.9977
P = 0.9609
P = 0.9271
Klebsiella relative abundance
100
50
0
K. pneumoniae
K. variicola
K. quasipneumoniae
b
P = 0.0087
Klebsiella relative abundance
100
75
50
25
0
+ high risk abx
- high risk abx
c
P < 0.0001
P = 0.0297
P = 0.0106
P = 0.5405
P = 0.2725
P = 0.7260
Klebsiella relative abundance
125
100
75
50
25
0
Case + high risk abx
Case - high risk abx
Control + high risk abx
Control - high risk abx
d
P value
aac(6')-Ib-cr5 36.29 17.20 0.0296
blaOXA-1 32.42 17.60 0.0680
blaCTX-M-15 28.15 17.76 0.1722
group_13461 22.70 18.14 0.5491
group_13458 24.75 18.06 0.4114
group_13460 23.01 18.14 0.5356
group_13437 20.69 18.23 0.7192
group_5875 19.57 18.36 0.8783
tehA_2 19.57 18.36 0.8783
group_10494 19.57 18.36 0.8783
tnpR 23.56 17.98 0.4140
blaTEM-1 26.45 17.55 0.1549
group_9575 29.53 16.91 0.0274
group_13467 24.03 17.76 0.2993
group_5455 11.20 18.86 0.3464
sul2 11.20 18.86 0.3464
group_13965 33.26 17.19 0.0210
group_9537 29.26 16.76 0.0226
group_13443 32.06 16.41 0.0047
group_7606 43.97 16.67 0.0003
dgcE 29.11 15.58 0.0029
aqpZ_2 35.11 15.57 0.0002
group_2409 28.97 16.43 0.0137
pdeC_2 27.83 15.58 0.0054
sul1 49.54 16.28 <0.0001
qacE1 46.51 16.35 <0.0001
arsD_1 32.89 14.66 <0.0001
group_774 26.18 14.73 0.0039
group_7231 23.21 17.25 0.2044
group_9530 21.09 17.22 0.3373
ltrA 25.23 15.56 0.0177
group_9528 25.09 16.51 0.0552
group_622 20.54 17.41 0.4331
group_4187 22.06 16.96 0.2169
umuD_2 19.62 17.50 0.5749
umuC_2 19.45 17.31 0.5696
group_2513 13.96 19.34 0.2825
Present
Absent
0
10
20
30
40
50
Klebsiella relative abundance (%)

Supplementary Figure S10. Gut relative abundance in rectal swabs from *Klebsiella* colonized patients.

a Comparison of *Klebsiella* gut relative abundance in patient rectal swabs stratified by *Klebsiella* species (mean ± SE displayed, Tukey's multiple comparisons test following ordinary one-way ANOVA). Sample sizes for *K. pneumoniae*, *K. variicola*, *K. quasipneumoniae* are n = 175, n = 42, and n = 15, respectively. **b** Comparison of *Klebsiella* gut relative abundance in patient rectal swabs between patients with and without a history of exposure to high-risk antibiotics ("abx," mean ± SE displayed, two-sided Student's t-test). Sample sizes for + high-risk abx and - high-risk abx are n = 60 and n = 170, respectively. **c** Comparison of *Klebsiella* gut relative abundance in patient rectal swabs stratified by patient case status and history of exposure to high-risk antibiotics ("abx," mean ± SE displayed, Tukey's multiple comparisons test following ordinary one-way ANOVA). Sample sizes for case + high-risk abx, case - high-risk abx, control + high-risk abx, and control - high-risk abx are n = 30, n = 53, n = 30, and n = 117, respectively. **d** Heatmap of mean *Klebsiella* gut relative abundance in patient rectal swabs stratified by gene presence/absence (bold *P* value indicates significant difference, two-sided Student's t-test). For **a**-**c**, each data point represents a single patient rectal swab. Source data are provided as a Source Data file.

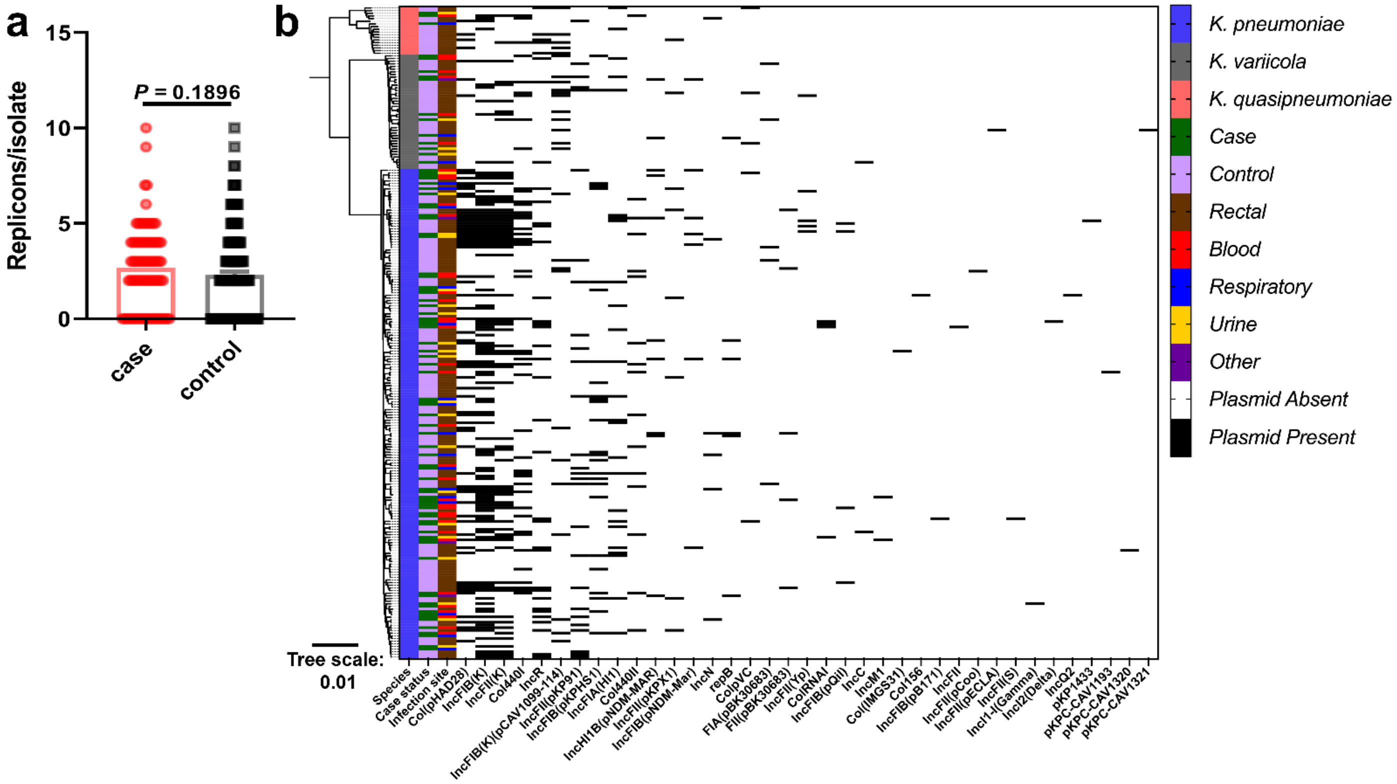

Supplementary Figure S11. Replicon presence in sequences *Klebsiella* isolates.

a Comparison of the number of replicons per isolates between cases (n = 85) and controls (n = 160, mean ± SE displayed, two-sided Student’s t-test). Each data point represents an individual isolate. **b** Heatmap of replicon presence/absence organized vertically by core genome phylogeny and horizontally by replicon type.

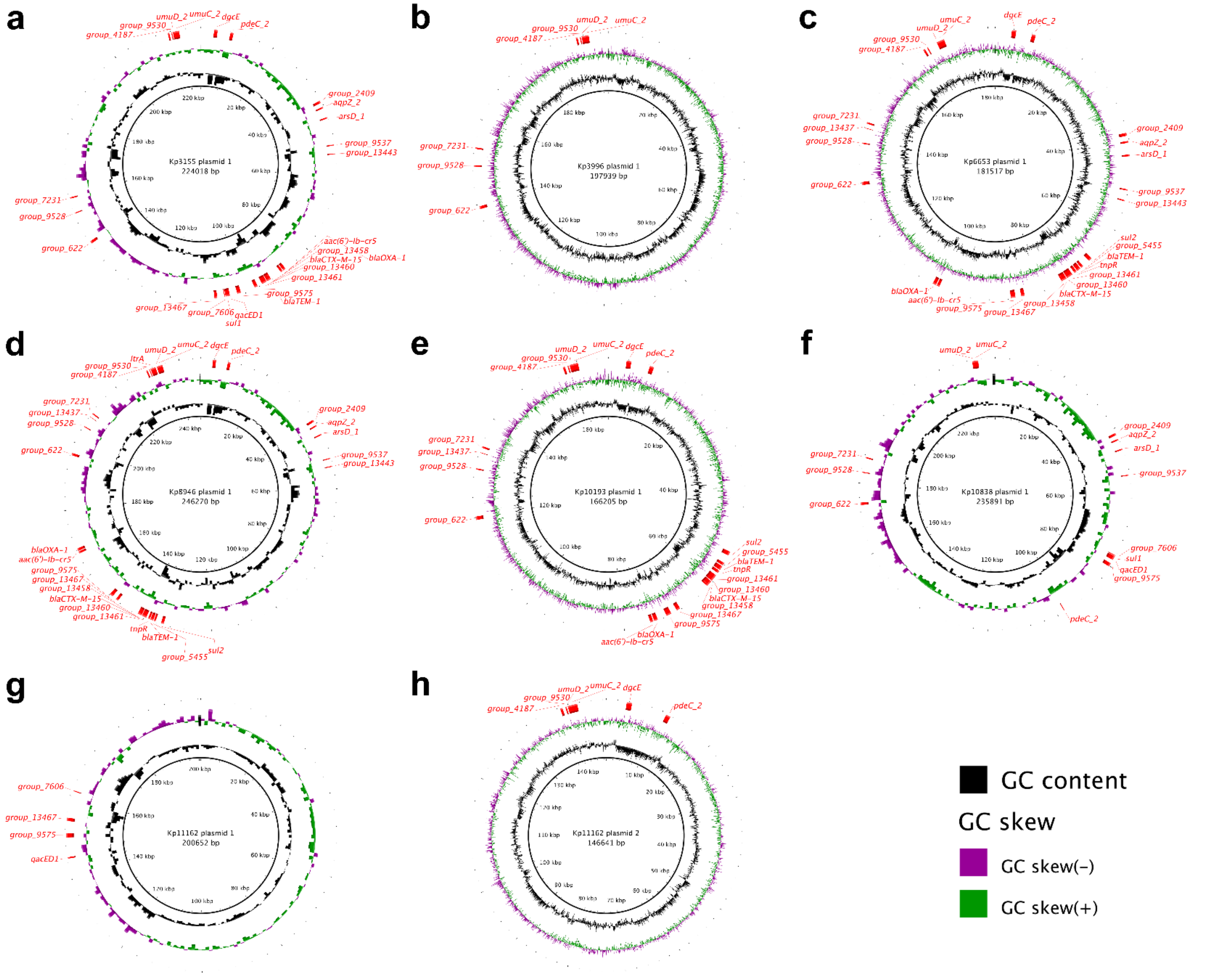

Supplementary Figure S12. Mapping of infection-associated genes to select *Klebsiella* plasmids

(**a**-**h**) Visualization of various hybrid assemblies of *Klebsiella* plasmids with mapped locations of infection-associated genes.

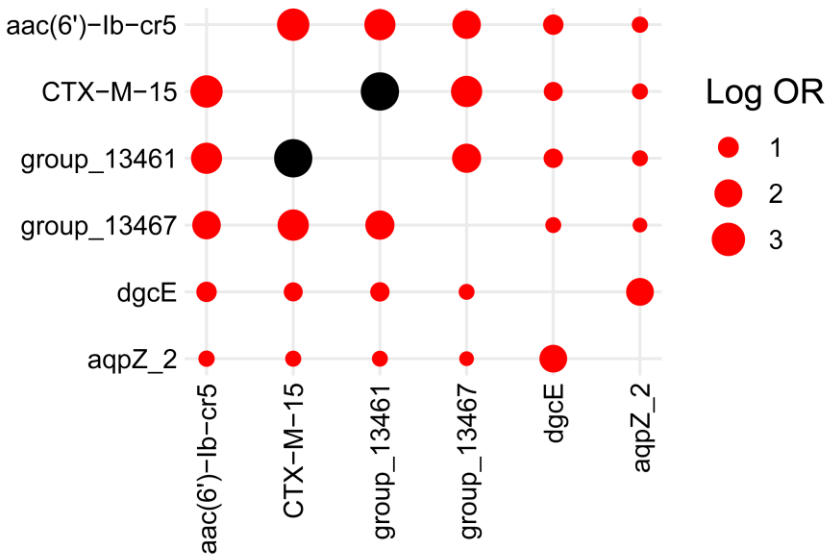

Supplementary Figure S13. Association between infection-associated genes in a geographically independent cohort of *Klebsiella* colonized patients

Odds ratios and Fisher *P* values (two-sided) were calculated between all pairs of infection-associated genes. Log ORs displayed in red have a Fisher *P* value ≤ 0.01, and those displayed in black have incalculable ORs due to identical presence/absence patterns.

Supplementary Table 1. Summary of treeWAS analysis

Locus	Terminal score	Terminal score *P* value	Simultaneous score	Simultaneous score *P* value	Subsequent score	Subsequent score *P* value
aac(6')-Ib-cr5	0.420408163	0	4	0.061764706	281.3333333	0
blaOXA-1	0.404081633	0	3	0.097058824	274	0
blaCTX-M-15	0.404081633	0	4	0.061764706	276.3333333	0
group_13461	0.404081633	0	3	0.097058824	277	0
group_13458	0.387755102	0	4	0.061764706	274.3333333	0
group_13460	0.395918367	0	3	0.097058824	276	0
group_13437	0.387755102	0	10	0	270.3333333	0
group_5875-tehA_2-group_10494	0.363265306	0	3	0.097058824	257	0
tnpR	0.387755102	0	3	0.097058824	272	0
blaTEM-1	0.404081633	0	3	0.097058824	277	0
group_9575	0.412244898	0	5	0.014705882	282.6666667	0
group_13467	0.404081633	0	5	0.014705882	284.6666667	0
group_5455-sul2	0.363265306	0	3	0.097058824	263	0
group_13965	0.363265306	0	2	0.202941176	263.6666667	0
group_9537	0.363265306	0	7	0	272.3333333	0
group_13443	0.363265306	0	7	0	278.3333333	0
group_7606	0.371428571	0	2	0.202941176	264.6666667	0
dgcE	0.363265306	0	8	0	247.6666667	0.005882353
aqpZ_2	0.355102041	0	7	0	274.3333333	0
group_2409	0.346938776	0	6	0.008823529	274	0
pdeC_2	0.33877551	0	8	0	232.6666667	0.058823529
sul1	0.363265306	0	1	0.438235294	264.3333333	0
qacED1	0.371428571	0	2	0.202941176	264.6666667	0
arsD_1	0.33877551	0	8	0	265.6666667	0
group_774	0.297959184	0.020588235	6	0.008823529	208	0.158823529
group_7231	0.33877551	0	10	0	258.3333333	0
group_9530	0.314285714	0.014705882	11	0	233.6666667	0.055882353
ltrA	0.306122449	0.017647059	6	0.008823529	206	0.158823529
group_9528	0.33877551	0	10	0	246.3333333	0.011764706
group_622	0.289795918	0.029411765	9	0	190	0.25
group_4187	0.314285714	0.014705882	9	0	241	0.035294118
umuD_2	0.240816327	0.129411765	9	0	115	0.591176471
umuC_2	0.183673469	0.282352941	1	0.438235294	50.33333333	0.902941176
group_2513	0.06122449	0.755882353	-1	0.438235294	117.6666667	0.582352941

Supplementary Table 2. *Klebsiella* phenotypic AMR association with infection

No adjustments were made for multiple comparisons.

Antibiotic	Case frequency	Control frequency	Unadjusted		Adjusted for clinical variables	
			Odds ratio	Fisher *P* value	Odds ratio	Fisher *P* value
Ampicillin	1.000	1.000	NA	NA	NA	NA
Chloramphenicol	0.107	0.069	1.96	0.173	1.31	0.662
Gentamicin	0.071	0.013	4.87	0.062	4.51	0.136
Ciprofloxacin	0.190	0.031	6.59	4.49E-04	7.78	1.66E-03
Cefazolin	0.262	0.044	4.48	5.78E-04	4.14	3.03E-03
Cefuroxime	0.224	0.063	7.15	2.13E-05	5.76	8.82E-04
Cefepime	0.214	0.031	7.7	1.19E-04	6.02	3.65E-03

Supplementary Table 3. Plasmid replicon associations with infection

No adjustments were made for multiple comparisons.

Replicon	Case frequency	Control frequency	Odds ratio	Fisher *P* value	Adjusted Odds ratio	Adjusted Fisher *P* value
Col(pHAD28)	0.305	0.287	1.09	0.77	1.05	0.877
IncFIB(K)	0.537	0.38	1.89	0.02	1.57	0.122
IncFII(K)	0.354	0.273	1.45	0.20	1.46	0.203
Col440I	0.22	0.167	1.41	0.32	1.34	0.398
IncR	0.11	0.173	0.59	0.20	0.66	0.330
IncFIB(K)(pCAV1099-114)	0.0976	0.127	0.75	0.51	0.64	0.341
IncFII(pKP91)	0.0732	0.133	0.51	0.17	0.61	0.336
IncFIB(pKPHS1)	0.146	0.0867	1.81	0.17	2.05	0.143
IncFIA(HI1)	0.0732	0.113	0.62	0.33	0.44	0.116
Col440II	0.122	0.04	3.33	0.02	3.03	0.090
IncHI1B(pNDM-MAR)	0.0732	0.0133	5.84	0.03	8.52	0.015
IncFII(pKPX1)	0.0122	0.0467	0.25	0.20	0.06	0.014

Supplementary Table 4. Primers and probes used in this study

Target	Forward primer	Reverse primer	Probe
bla$_{CTX\text{-}M\text{-}15}$	GCAGATAATTCGCAAA TACTTTATCGTGCTGA	TGACGTGCTTTTCCGC AATCGGATTATAGTTA	{ABY}AGCGCTTTGCGATGTGCAGCA CCAGTAAAGT{QSY-7}
aqpZ_2	ATTCTGATTATGAGCAT CCTAACGACCAGA	AGCGGCCCAGTACAGC CACTGATT	{VIC}TAGCTTCGGGCCGGCATTGGC CATGGGATACT{QSY-7}
group_13461	AAGGTTCCGAATACGA CTACTTTTTCTTTGTA	TGAAACACGGCTTCAT TCGCCCAAAA	{ABY}AGAGATGACCCACCGCCATGT CGTATTTGG{QSY-7}
group_13467	GCCTGCCACGCTCAAT ACCGACAAA	CGCGCACCGGCTTGAT CAGTATCTT	{VIC}CCGAGCTATGGTGCAGCGATCA CCGAATT{QSY-7}
aac(6')-Ib-cr5	GACCAGTTACTGGCGA ATGCATCACAA	GCCTCTCAAACCCCGC TTTCTCGTA	{VIC}CTTGGTGACCTCGGGATCATTG AACA GCAACT{QSY-7}
dgcE	GTTATGGGCTGAAGAT AAAAGAGACAGATAGA	GCAGTCTTACGGGATG CCCTGCATA	{ABY}AGTGCCCGATGCAAGCCTTGA TCCA AGATT{QSY-7}
fiu	AACGTAGCGCAGGATG GATCTTCCG	GACAGATCGCTGGTGG CCTGATA	{FAM}CGTCCACAGCGTAAAGGCATG TT{MGB]
