# Supplementary material for: Combined comparative genomics and clinical modeling reveals plasmid-encoded genes are independently associated with Klebsiella infection
Source: Nat Commun. 2022 Aug 1;13:4459. doi: 10.1038/s41467-022-31990-1 (PMC9343666; doi:10.1038/s41467-022-31990-1)
Supplement: Supplementary file 3 — Description of Additional Supplementary Files [file 41467_2022_31990_MOESM3_ESM.pdf]

Description of additional supplementary files

File Name: Supplementary Data 1 Description: Klebsiella genome assembly details

File Name: Supplementary Data 2 Description: Klebsiella isolate gene presence/absence

File Name: Supplementary Data 3 Description: Unit association with case status

File Name: Supplementary Data 4 Description: Klebsiella genotyping with Kleborate

File Name: Supplementary Data 5 Description: Klebsiella rectal isolate genotyping with PlasmidFinder
