# Supplementary material for: Combined comparative genomics and clinical modeling reveals plasmid-encoded genes are independently associated with Klebsiella infection
Source: Nat Commun. 2022 Aug 1;13:4459. doi: 10.1038/s41467-022-31990-1 (PMC9343666; doi:10.1038/s41467-022-31990-1)
Supplement: Supplementary file 9 — Reporting Summary [file 41467_2022_31990_MOESM9_ESM.pdf]

nature portfolio

Corresponding author(s): Michael A. Bachman

Last updated by author(s): May 27, 2022

Reporting Summary

Nature Portfolio wishes to improve the reproducibility of the work that we publish. This form provides structure for consistency and transparency in reporting. For further information on Nature Portfolio policies, see our Editorial Policies and the Editorial Policy Checklist.

Statistics

For all statistical analyses, confirm that the following items are present in the figure legend, table legend, main text, or Methods section.

n/a	Confirmed	
☐	☒	The exact sample size (*n*) for each experimental group/condition, given as a discrete number and unit of measurement
☐	☒	A statement on whether measurements were taken from distinct samples or whether the same sample was measured repeatedly
☐	☒	The statistical test(s) used AND whether they are one- or two-sided<br>*Only common tests should be described solely by name; describe more complex techniques in the Methods section.*
☐	☒	A description of all covariates tested
☐	☒	A description of any assumptions or corrections, such as tests of normality and adjustment for multiple comparisons
☐	☒	A full description of the statistical parameters including central tendency (e.g. means) or other basic estimates (e.g. regression coefficient) AND variation (e.g. standard deviation) or associated estimates of uncertainty (e.g. confidence intervals)
☐	☒	For null hypothesis testing, the test statistic (e.g. *F*, *t*, *r*) with confidence intervals, effect sizes, degrees of freedom and *P* value noted<br>*Give P values as exact values whenever suitable.*
☒	☐	For Bayesian analysis, information on the choice of priors and Markov chain Monte Carlo settings
☒	☐	For hierarchical and complex designs, identification of the appropriate level for tests and full reporting of outcomes
☐	☒	Estimates of effect sizes (e.g. Cohen's *d*, Pearson's *r*), indicating how they were calculated

Our web collection on statistics for biologists contains articles on many of the points above.

Software and code

Policy information about availability of computer code

Data collection	No software was used for data collection.
Data analysis	All code developed for this manuscript is available at https://github.com/rdcrawford/Bachman_CU8.<br><br>All other analysis software (Trimmomatic v0.39, SPAdes v3.13.0, Prokka v1.14.6, Roary v3.13.0, Kleborate v1.0.0, PlasmidFinder v2.0.1, NanoFilt v2.5.0, Unicycler v0.4.8, BLAST v2.9.0, MAFFT v7.310, fastTree v2.1.10, APE R package v5.3, iNEXT R package v2.0.20, BRIG v0.95, Scoary v1.6.16, treeWAS v1.1, survey R package v4.1-1, mediation R package v4.5.0, lme4 R package v1.1-29, R Studio v1.2.5001, and R versions 3.6.3 and v4.0.5) is publicly available and references appropriately in the methods section.

For manuscripts utilizing custom algorithms or software that are central to the research but not yet described in published literature, software must be made available to editors and reviewers. We strongly encourage code deposition in a community repository (e.g. GitHub). See the Nature Portfolio guidelines for submitting code & software for further information.

Data

Policy information about availability of data

All manuscripts must include a data availability statement. This statement should provide the following information, where applicable:

- Accession codes, unique identifiers, or web links for publicly available datasets
- A description of any restrictions on data availability
- For clinical datasets or third party data, please ensure that the statement adheres to our policy

The sequencing data generated in this study have been deposited in the Sequence Read Archive (SRA) database under accession code PRJNA789565. The human subjects data are available under restricted access for the protection of the rights and welfare of human research subjects. Access to deidentified data can be

obtained from MAB within one year upon request, pending approval from the University of Michigan Institutional Review Board. The remaining data generated in this study are provided in the Supplementary Information/Source Data file.

Field-specific reporting

Please select the one below that is the best fit for your research. If you are not sure, read the appropriate sections before making your selection.

☒ Life sciences ☐ Behavioural & social sciences ☐ Ecological, evolutionary & environmental sciences

For a reference copy of the document with all sections, see nature.com/documents/nr-reporting-summary-flat.pdf

Life sciences study design

All studies must disclose on these points even when the disclosure is negative.

Sample size	We originally calculated the ORs (>1.95) that 400 patients (100 cases and 3 controls are matched for each case, N=100, M=3) can detect with 80% power and 5% type I error (alpha), using the Matched Case-Control test. Case definition and consolidation led to an N = 85, and matching feasibility led to a 1:2, rather than a 1:3 matching scheme.
Data exclusions	The only data exclusions were WGS samples that did not correspond to members of the Klebsiella pneumoniae species complex, which is the focal point of analyses in this manuscript. Of the original 340 WGS samples, 10 were excluded.
Replication	WGS findings were reproduced in a second, geographically independent cohort of patients colonized by a member of the Klebsiella pneumoniae species complex. All experimental replicates shown represent at least 3 biological replicates.
Randomization	Randomization is not relevant to this study, as "cases" were defined as patients colonized by a member of the Klebsiella pneumoniae species complex that acquired a subsequent infection with that colonizing isolate. "Controls" were defined as patients colonized by a member of the Klebsiella pneumoniae species complex that remained asymptomatic and were matched 2:1 to cases based on clinical variables. Clinical covariates were selected based on a previous study (https://doi.org/10.1128/mSphere.00132-21) and controlled for using Inverse Probability Treatment Weighting.
Blinding	Blinding was not possible in this study, as WGS sample selection was based on determination of infection following extensive chart review and corresponding matching based on clinical variables.

Reporting for specific materials, systems and methods

We require information from authors about some types of materials, experimental systems and methods used in many studies. Here, indicate whether each material, system or method listed is relevant to your study. If you are not sure if a list item applies to your research, read the appropriate section before selecting a response.

Materials & experimental systems

n/a	Involved in the study	
☒	☐	Antibodies
☒	☐	Eukaryotic cell lines
☒	☐	Palaeontology and archaeology
☒	☐	Animals and other organisms
☐	☒	Human research participants
☒	☐	Clinical data
☒	☐	Dual use research of concern

Methods

n/a	Involved in the study	
☒	☐	ChIP-seq
☒	☐	Flow cytometry
☒	☐	MRI-based neuroimaging

Human research participants

Policy information about studies involving human research participants

Population characteristics	Population characteristics are described in Table 1 of this manuscript.
Recruitment	This is a retrospective data review study without direct involvement of human subjects. Patient recruitment is described elsewhere (https://doi.org/10.1128/mSphere.00132-21). Per this study: "We sequentially enrolled subjects to establish a cohort of patients with Klebsiella (K. pneumoniae or K. variicola) rectal colonization. Per institutional protocols, patients are screened for vancomycin-resistant Enterococcus (VRE) by rectal swab upon admission to the intensive care units (ICUs) and the oncology wards of the University of Michigan hospitals in Ann Arbor, MI. From May 2017 to September 2018, we considered patients eligible for inclusion based on the submission of a rectal swab for a VRE screening culture. All patients admitted to these wards undergo screening for rectal VRE carriage per hospital policy, using a flocked swab placed into Amies transport medium (ESwab; Becton, Dickinson, Franklin Lakes, NJ). Subjects were subsequently enrolled in our study if K. pneumoniae was isolated from the swab media through plating on MacConkey agar followed by taxonomic identification using matrix-assisted laser desorption ionization–time of flight (MALDI-TOF) mass spectrometry." This study was performed with a waiver of informed consent since the research involves no more than minimal risk to the subjects and could not practicably be carried out without the waiver or alteration. Patient recruitment was identical at both recruitment sites.
Ethics oversight	Patient enrollment and sample collection at the University of Michigan was approved by and performed in accordance with the Institutional Review Boards (IRB) of the University of Michigan Medical School (Study number HUM00123033). Patient enrollment and sample collection at Johns Hopkins University was approved and performed in accordance with the IRB of the Johns Hopkins University (Study number IRB00129775).

Note that full information on the approval of the study protocol must also be provided in the manuscript.
